# Effects of *Clonorchis sinensis* combined with Hepatitis B virus infection on the prognosis of patients with Hepatocellular Carcinoma following Hepatectomy

**DOI:** 10.1371/journal.pntd.0011012

**Published:** 2023-01-13

**Authors:** Yuan-Kuan Li, Jing-Fei Zhao, Cheng-Lei Yang, Guo-Hua Zhan, Jie Zhang, Shang-Dong Qin, Min Zhou, Min-Jun Li, Jun-Tao Huang, Feng-Yao Kong, Hai Huang, Jia-Hao Chen, Bang-De Xiang

**Affiliations:** 1 Department of Hepatobiliary Surgery, Guangxi Medical University Affiliated Tumor Hospital, Guangxi, China; 2 Department of Hepatobiliary Surgery, Guangxi Medical University Affiliated Wuming Hospital, Guangxi, China; Instituto de Salud Carlos III, SPAIN

## Abstract

**Background:**

This study aimed to determine the impact of co-infection of *Clonorchis sinensis* (CS) and hepatitis B virus (HBV) on the prognosis of patients with hepatocellular carcinoma (HCC) following hepatectomy.

**Methods:**

The clinicopathological information of 946 patients with HCC following hepatectomy was retrospectively analyzed. The patients were divided into four groups depending on whether they had CS infection and/or HBV infection: double-negative group (infected with neither CS nor HBV), simple CS group (infected with only CS), simple HBV group (infected with only HBV), and double-positive group (co-infected with CS and HBV). Kaplan-Meier curves were used to evaluate the overall survival (OS) and recurrence-free survival (RFS), while log-rank tests were used to compare survival rates. Further, Cox regression was used to perform both univariate and multivariate survival analyses to identify variables linked to the prognosis of HCC.

**Results:**

The median overall survival (OS) and recurrence-free survival (RFS) in the double-positive, simple CS, simple HBV, and double-negative groups were 27 months and 9 months, 20 months and 7 months, 44 months and 12 months, and 42 months and 17 months, respectively. The double-positive group’s 1-year, 3-year, and 5-year OS and RFS rates were 79.2% and 46.9%, 62.6% and 28.4%, 47.8%, and 12.2%, respectively. The simple CS group’s 1-year, 3-year, and 5-year OS and RFS rates were 86.3% and 41.5%, 56.5% and 27.7%, 50.2%, and 18.5%, respectively. The simple HBV group’s 1-year, 3-year, and 5-year OS and RFS rates were 89.8% and 56.0%, 72.5% and 30.5%, 63.8%, and 19.9%, respectively. The double-negative group’s 1-year, 3-year, and 5-year OS and RFS rates were 91.5% and 62.3%, 76.1% and 32.9%, 64.0%, and 22.4%, respectively. Further, according to a Cox multivariate analysis, tumor size (> 5cm), Edmonson grade (III-IV), BCLC-C stage, and tumor satellite focus were independent risk factors for RFS and OS in patients with HCC.

**Conclusion:**

Patients with HCC and *Clonorchis sinensis* infection experience a poor prognosis after hepatectomy, regardless of whether they are co-infected with HBV.

## Author summary

*Clonorchis sinensis* inhabits the bile ducts of mammals, and clonorchiasis is a major public health concern in numerous countries. Infection with Clonorchis sinensis is a risk factor for cholangiocarcinoma. It is unknown whether it changes the prognosis of hepatocellular carcinoma patients or contributes to this disease’s development. The hepatitis B virus is the leading cause of hepatocellular carcinoma. In addition, hepatitis B infection is prevalent in many endemic places for clonorchiasis, although the potential interaction between the two diseases has gotten little attention. This study aimed to determine the prognostic significance of *Clonorchis sinensis* infection and *Clonorchis sinensis* combined with HBV infection in patients with hepatocellular carcinoma after hepatectomy. Our results indicate that patients with hepatocellular carcinoma combined with *Clonorchis sinensis* infection have a worse prognosis after hepatectomy with or without HBV infection, providing evidence for future studies on the adverse effects of the interaction between the two on patients with hepatocellular carcinoma.

## Introduction

Primary liver cancer is the seventh most common malignancy and the second most common cause of death in patients with malignancies worldwide [1]. Hepatocellular carcinoma (HCC) is the most common histological type of liver cancer that makes up about 75% of all cases of liver cancer [2]. Although there are multiple options for treating HCC, the prognosis is still poor due to the disease’s late diagnosis and the high recurrence rate following surgery [3,4]. Surgical resection remains the most effective treatment for HCC [5,6].

The hepatitis B-causing pathogen is the hepatitis B virus (HBV), a member of the Hepadnaviridae family. Around 240 million people worldwide have chronic hepatitis B (CHB) virus infection, significantly increasing their risk of developing cirrhosis, liver failure, and hepatocellular carcinoma in their lifetime [7]. Chronic hepatitis B infection is one of the leading causes of HCC [8], and studies have shown that HBV infection significantly impacts patients’ prognoses [9,10].

*Clonorchis sinensis* (CS) is a parasite that resides in the bile ducts of mammals. Freshwater fish are intermediate hosts for the parasite, and its infection leads to clonorchiasis, widespread in eastern Russia, the Korean Peninsula, northern Vietnam, and parts of China [11]. More than 15 million people worldwide have received a CS infection diagnosis [12]. While another 200 million people are at risk of contracting the parasite [13]. Guangdong and Guangxi, in the southeast, and Heilongjiang, Jilin, and Liaoning, in the northeast, are China’s two most endemic regions for CS; the prevalence of CS infection in male individuals is usually higher than that in female individuals [12,14]. According to the most recent parasitological epidemiological study in China, performed between 2014 and 2016, the weighted infection rate of CS was 0.47%, and the number of CS infections in the country was around 5.98 million [15]. The association between CS infection and cholangiocarcinoma is well established, and CS has been classified as a class I carcinogen that could induce cholangiocarcinoma [16]. Studies have shown that CS infection or CS combined with HBV infection are important factors that cause Intrahepatic cholangiocarcinoma and HCC [17].

Further, the risk factors affecting the occurrence of tumors may also affect the prognosis of patients. However, no study has reported an association between CS infection and recovery from hepatectomy in patients with HCC. This study examines the prognostic significance of CS infection and CS paired with HBV infection in patients with HCC following hepatectomy.

## Patients and methods

### Ethics statement

The Ethics Committee of the Affiliated Cancer Hospital of Guangxi Medical University and the Ethics Committee of the Affiliated Wuming Hospital of Guangxi Medical University approved the study protocol. On admission, all patients provided written consent for their anonymized medical data to be analyzed and published for research purposes.

### Study population and data collections

**Fig 1:**
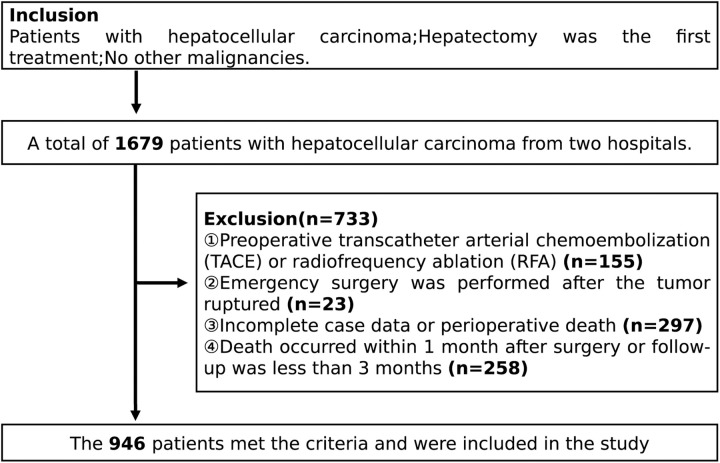
The flow of study participant.

Out of a total of 1679 available patients, 1644 suffered from hepatocellular carcinoma that underwent liver resection at the Guangxi Medical University Affiliated Cancer Hospital between January 2012 and December 2017, while 35 patients suffering from hepatocellular carcinoma combined with CS infection underwent liver resection at the Guangxi Medical University Affiliated Wuming Hospital between January 2011 to May 2021. The procedure for including and excluding participants from this study is demonstrated in Fig 1.

Following were the Inclusion criteria: (1) Hepatectomy with a postoperative pathological diagnosis of hepatocellular carcinoma; (2) Hepatectomy was the first treatment, and there was no history of other malignant tumors. The following criteria led to the exclusion of 733 patients from the study: (1) 155 patients underwent radiofrequency ablation (RFA) or transcatheter arterial chemoembolization (TACE) prior to surgery, and (2) 23 patients underwent emergency surgery for tumor rupture; (3) 297 patients had incomplete case data, or patients died perioperatively; (4) 258 patients either passed away one month after surgery or had less than three months of follow-up. Based on these criteria, 946 patients were included in this study.

Following were the diagnostic criteria for Clonorchiasis and HBV. Diagnostic criteria for clonorchiasis: Only one of the following conditions was considered sufficient to confirm the diagnosis of Clonorchis Sinensis [18–22]. (1) Clinical diagnosis cases: Epidemiological history of *Clonorchis sinensis* (the patient should have a medical history of eating raw or half-raw freshwater fish or shrimp, and a history of living, working, and traveling in endemic areas) and the presence of a mild dilatation of the intrahepatic bile ducts, detected by imaging (MRI, CT, or ultrasonography determined the bile duct dilatation pattern). Local dilatation caused by tumor compression, bile duct stones, thrombosis, or cancerous embolism was not considered; (2) Intraoperative or postoperative pathological examination reveals the presence of adult *Clonorchis sinensis* in the liver or gallbladder; (3) Preoperative fecal examination was positive for eggs of *Clonorchis sinensis*.

HBV diagnostic criteria: The patient’s preoperative serum was positive for hepatitis B surface antigen (qualitative) [23].

A retrospective analysis of patient data included in this study was performed. The data collection included the following: (1) General information: gender, age, the experience of eating raw fish; (2) Hematological examination: tumor markers, alpha-fetoprotein(AFP), hepatitis B surface antigen, total bilirubin, complete blood count, albumin, aspartate aminotransferase (AST), alanine aminotransferase (ALT); (3) Imaging examination: bile duct dilatation; (4) Pathological indicators: liver cirrhosis, number of tumors, tumor length, and degree of tumor differentiation (based on the Edmondson-Steiner criteria for tumor histological differentiation: Grade I, well-differentiated; Grade II, moderately differentiated; Grade III-IV, poorly differentiated), tumor capsule, macrovascular invasion, and microvascular invasion (MVI), tumor satellite focus; (5) Other examinations: Intraoperative CS examination and fecal examination.

### Hepatectomy

All surgical procedures were performed by specialists qualified in hepatectomy at the Cancer Hospital and Wuming Hospital, affiliated with Guangxi Medical University. All patients underwent surgery using standard techniques. The initial treatment was radical resection, which involves the elimination of all malignant lesions based on visual inspection and a negative histological margin.

### Postoperative treatment

Postoperative treatment of HCC was performed to reduce the recurrence rate. The patients with HBV-infected HCC underwent postoperative long-term antiviral therapy with nucleoside analogs. The HCC patients with combined large-vessel carcinoma thromboses, such as a portal or hepatic vein, were treated with postoperative prophylactic TACE. The patients with recurrent HCC were treated with secondary surgical resection, TACE, radiotherapy, and targeted immunotherapy in accordance with the form of recurrence. Anthelmintic therapy was not administered to HCC patients with CS before or after surgery, and deworming was only done if the patient volunteered for anthelmintic therapy.

### Follow-up routine

Regular postoperative reviews and phone calls by professional staff were done to monitor the patients for cases of recurrence and the time of it. The date and reason for death were also recorded in case of fatalities. Tumor recurrence was diagnosed with the imaging manifestations of CT or MRI. Intrahepatic recurrence was diagnosed *via* imaging only if the tumor exhibited typical enhancing features. The biopsy confirmed extrahepatic tumors or tumors with atypical imaging features for HCC. All patients were followed up once every month for three months following surgery and then once every three months after that. After two years, patients were followed up once every six months. A blood routine, liver function test, serum alpha-fetoprotein, abdominal ultrasound examination, and a CT or MRI were conducted as a part of the routine follow-up. Overall survival (OS) was determined to be the interval between the surgery date and the patient’s HCC-related death or the last follow-up. Recurrence-free survival (RFS) was determined as the interval between the surgery date and the patient’s tumor recurrence or the date of the last follow-up. The follow-up deadline for this study was May 31, 2020.

### Statistical analysis

SPSS Version 22.0 (SPSS Inc, Chicago, Illinois, USA) for Windows was used for the statistical analysis. The normally distributed measurement data were presented as mean ± standard deviation, and the independent samples were subjected to a t-test or ANOVA to investigate group differences. The rank-sum test was used to determine group differences from non-normally distributed measurement data expressed as medians. Further, Fisher’s exact or chi-square tests were employed to compare group differences in categorical data, expressed as rates. Plotting survival curves and survival analysis were done using the Kaplan-Meier method. The cumulative OS and RFS rates were evaluated using curves and log-rank tests to compare between groups. Cox regression models were used for univariate and multifactorial prognostic analyses. To determine independent risk factors for OS and RFS, variables with statistically significant differences (P < 0.05) in univariate analysis were included in multivariate Cox analysis. A P-value < 0.05 was defined to be statistically significant.

## Results

### Characteristics of the Study Population

A total of 946 patients were enrolled in the study, including 820 males and 126 females, with an average age of (51±10) years. The patients were split into a CS-positive (with CS infection) and a CS-negative group (without CS infection). Additionally, all patients were divided into HBV-positive and HBV-negative groups based on the presence or absence of preoperative serum hepatitis B surface antigen. Table 1 provides detailed clinical baseline data for patients in each group. Patients with HCC in the CS-positive group displayed statistically significant differences from the CS-negative group (P<0.05, Table 1) in gender, tumor size, MVI, BCLC stage, macrovascular invasion, liver cirrhosis, albumin, absolute neutrophil count (NEUT), the absolute number of eosinophils (EO), and absolute lymphocyte count (LYMPH). Comparing the HBV-positive and HBV-negative groups reveals statistically significant differences in age, cirrhosis, ALT, AST, and AFP (P 0.05, Table 1).

**Table 1:** Clinicopathology of patients with hepatocellular carcinoma treated by hepatic resection.

Variable	All patients (N = 946)		P	All patients (N = 946)		P
	CS- positive (N = 204)	CS- negative (N = 742)		HBV- positive (N = 789)	HBV- negative (N = 157)	
Gender			<0.001			0.427
Male	198	622		687	133	
Female	6	120		102	24	
Age			0.174			<0.001
<60y	163	559		635	87	
≥60y	41	183		154	70	
Tumor size			0.023			0.806
≤5cm	72	328		335	65	
˃5cm	132	414		454	92	
NO. of tumors			0.939			0.442
<2	146	529		559	116	
≥2	58	213		230	41	
Capsule of tumor			0.937			0.643
NO	41	151		158	34	
Yes	163	591		631	123	
MVI			0.001			0.651
Negative	99	452		457	94	
Positive	105	290		332	63	
BCLC stage			<0.001			0.306
A-B	127	567		584	110	
C	77	175		205	47	
Edmonson grade			0.458			0.309
I-II	93	360		372	81	
III-IV	111	382		417	76	
satellite focus			0.577			0.947
No	176	651		690	137	
Yes	28	91		99	20	
Macarovascular invasion			0.012			0.400
No	159	633		657	135	
Yes	45	109		132	22	
Liver cirrhosis			0.013			0.007
No	75	345		335	85	
Yes	129	397		454	72	
Serum albumin			0.026			0.353
<35g/L	33	78		96	15	
≥35g/L	171	664		693	142	
ALT			0.254			0.002
≤40U/L	122	476		482	116	
˃40U/L	82	266		307	41	
AST			0.896			0.023
≤40U/L	107	393		404	96	
˃40U/L	97	349		385	61	
TBil			0.109			0.831
≤17.1μmol/ml	157	608		639	126	
˃17.1μmol/ml	47	134		150	31	
AFP			0.116			0.019
<400 ng/ml	104	424		427	101	
≥400 ng/ml	100	318		362	56	
NEUT			<0.001			0.503
<3.82 x10^9^/L	61	408		395	74	
≥3.82 x10^9^/L	143	334		394	83	
LYMPH			0.002			0.066
<1.835 x10^9^/L	82	391		405	68	
≥1.835 x10^9^/L	112	351		384	89	
EO			<0.001			0.704
<0.2 x10^9^/L	46	413		385	74	
≥0.2 x10^9^/L	158	329		404	83	

BCLC: Barcelona Clinic Liver Cancer Staging System. MVI: Microvascular invasion. HBsAg: hepatitis B surface antigen. TBil: total bilirubin. AST: aspartate aminotransferase. ALT: alanine aminotransferase. NEUT: absolute neutrophil count. EO: the absolute number of eosinophils. LYMPH: absolute lymphocyte count. CS: Clonorchis sinensis. HBV: Hepatitis B Virus.

Depending on whether they had both CS and HBV infection, the 946 patients with HCC were further classified into four groups: double-negative group (both CS and HBV-negative, n = 119); simple CS group (CS-positive and HBV-negative, n = 38); simple HBV group (CS-negative and HBV-positive, n = 623); and double-positive group (both CS and HBV-positive, n = 166). The detailed clinical baseline information for the four groups of patients is shown in Table 2. The four groups were statistically different in gender, age, tumor size, MVI, BCLC stage, cirrhosis, albumin, ALT, AFP, NEUT, EO, and LYMPH (P<0.05, Table 2).

**Table 2:** Comparison of preoperative clinicopathological data of 4 groups of patients with hepatocellular carcinoma.

Variable	CS-negative and HBV-negative (N = 119)	CS-positive and HBV-negative (N = 38)	CS-negative and HBV-positive (N = 623)	CS-positive and HBV-positive (N = 166)	P
Gender					<0.001
Male	97	36	525	162	
Female	22	2	98	4	
Age					<0.001
<60y	68	19	491	144	
≥60y	51	19	132	22	
Tumor size					0.020
≤5cm	57	8	271	64	
˃5cm	62	30	352	102	
NO. of tumors					0.139
<2	83	33	446	113	
≥2	36	5	177	53	
Capsule of tumor					0.771
No	24	10	127	31	
Yes	95	28	496	135	
MVI					0.011
Negative	77	17	375	82	
Positive	42	21	248	84	
BCLC stage					0.001
A-B	86	24	481	103	
C	33	14	142	63	
Edmonson grade					0.632
I-II	63	18	297	75	
III-IV	56	20	326	91	
satellite focus					0.952
No	104	33	547	143	
Yes	15	5	76	23	
Macarovascular invasion					0.063
No	104	31	529	128	
Yes	15	7	94	38	
Liver cirrhosis					0.001
No	64	21	281	54	
Yes	55	17	342	112	
Serum albumin					0.002
<35g/L	5	10	73	23	
≥35g/L	114	28	550	143	
ALT					0.013
≤40U/L	89	27	387	95	
˃40U/L	30	11	236	71	
AST					0.115
≤40U/L	75	21	318	86	
˃40U/L	44	17	305	80	
TBil					0.459
≤17.1μmol/ml	97	29	511	128	
˃17.1μmol/ml	22	9	112	38	
AFP					0.041
<400 ng/ml	79	22	345	82	
≥400 ng/ml	40	16	278	84	
NEUT					<0.001
<3.82x10^9^/L	62	12	346	49	
≥3.82x10^9^/L	57	26	277	117	
LYMPH					0.005
<1.835x10^9^/L	55	13	336	69	
≥1.835x10^9^/L	64	25	287	97	
EO					<0.001
<0.2x10^9^/L	66	8	347	38	
≥0.2x10^9^/L	53	30	276	128	

BCLC: Barcelona Clinic Liver Cancer Staging System. MVI: Microvascular invasion. HBsAg: hepatitis B surface antigen. TBil: total bilirubin. AST: aspartate aminotransferase. ALT: alanine aminotransferase. NEUT: absolute neutrophil count. EO: the absolute number of eosinophils. LYMPH: absolute lymphocyte count. CS: Clonorchis sinensis. HBV: Hepatitis B Virus.

### Follow-up and prognosis

The enrolled patients had an RFS follow-up period of 1 to 98 months, with a median of 12 months. OS was followed up for 2 to 98 months, with a median of 40 months. 642 individuals experienced tumor recurrence during the follow-up period, and 331 patients passed away from hepatocellular carcinoma. OS and RFS for the patients in the CS, HBV, and four subgroups were evaluated.

**Fig 2:**
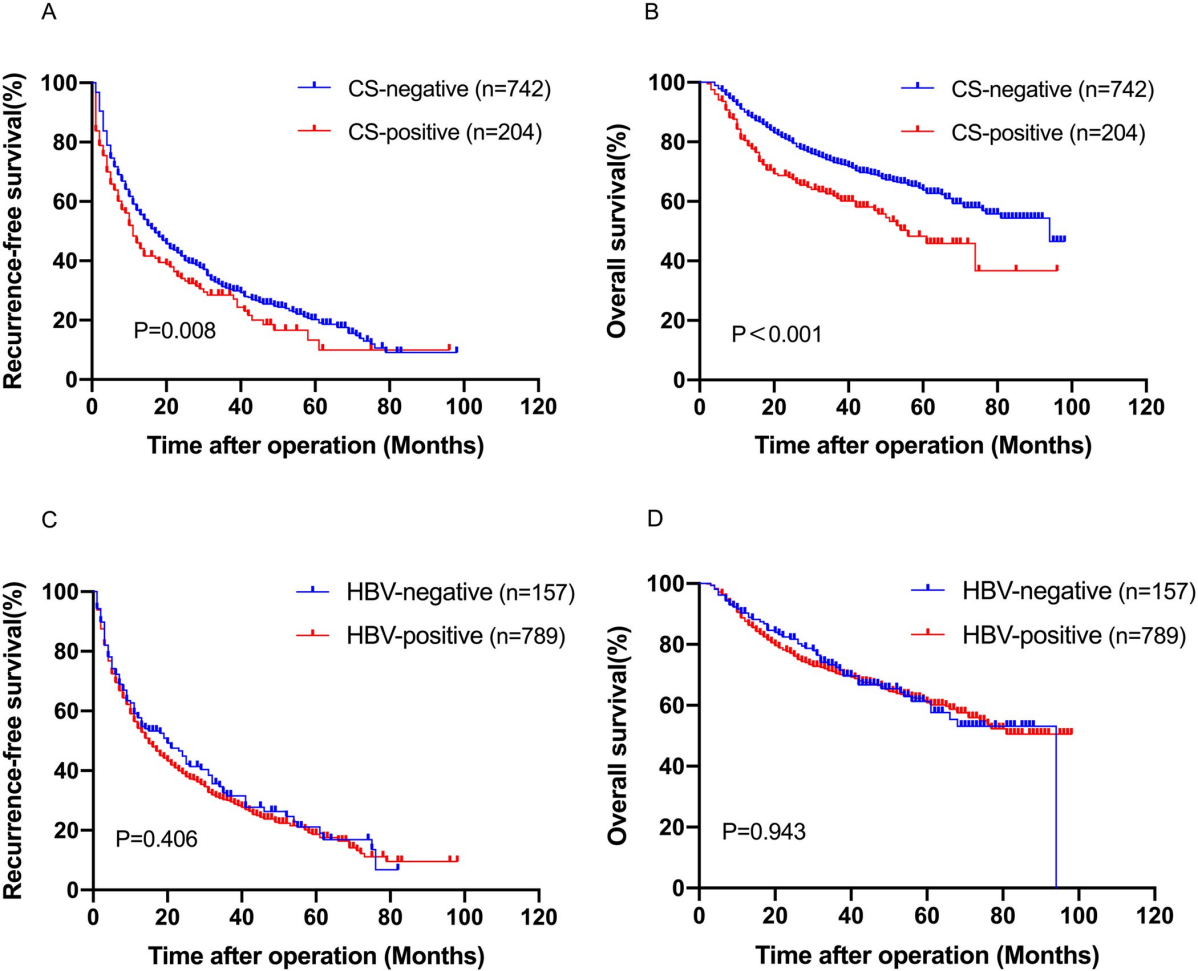
The influence of CS and HBV on the prognosis of patients with HCC after hepatectomy. (A), CS for recurrence-free survival; (B), CS for overall survival; (C), HBV for recurrence-free survival; (D), HBV for overall survival.

505 recurrences and 250 deaths were recorded in the CS-negative group, while 137 recurrences and 81 deaths were recorded in the CS-positive group. The cumulative survival rates in the CS-negative group were 90.1%, 73.1%, and 63.8% at 1, 3, and 5 years respectively, following surgery. These differences were statistically significant from 80.4%, 61.8%, and 48.3% in the CS-positive group (P<0.05). The median OS time in the CS-negative and the CS-positive groups was 43 months and 26 months, respectively. The cumulative recurrence-free survival rates in the CS-negative group were 57.0%, 30.8%, and 20.2% at 1, 3, and 5 years respectively, following surgery. These differences were statistically significant from 46.0%, 28.5%, and 13.3% in the CS-positive group (P<0.05). The median RFS time in the CS-negative and the CS-positive groups was 13 months and 8 months, respectively. The data analysis revealed that patients of the CS-positive group had a considerably worse prognosis than patients of the CS-negative group (5-year OS: 48.3% vs. 63.8%, P < 0.001; 5-year RFS: 13.3% vs. 20.2%, P = 0.008; Fig 2).

105 relapses and 54 deaths were recorded in the HBV-negative group, and 537 relapses and 277 deaths in the HBV-positive group. The median survival time of the HBV-negative and the HBV-positive groups was 37 and 41 months, respectively. In the two groups mentioned above, the median RFS time was 13 months and 12 months, respectively. However, the data showed no statistically significant differences in 5-year OS (60.9% versus 61.3%, P = 0.943, Fig 2) and RFS (18.6% versus 21.1%, P = 0.406, Fig 2) between HBV-positive and HBV-negative patients.

**Fig 3:**
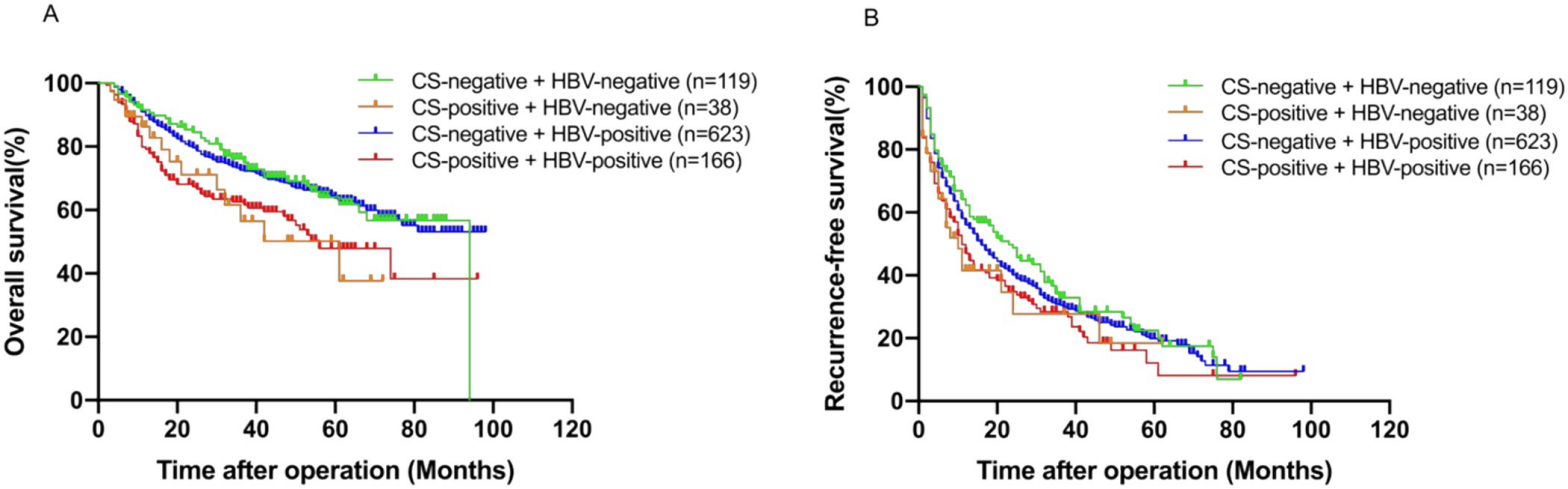
Comparison of OS and RFS among patients of groups 1 to 4. (A) The OS rates (P1–2 = 0.052, P2–3 = 0.05, P1–3 = 0.863, P3–4 < 0.001, P1–4 = 0.012, P2–4 = 0.924); (B) the RFS rates (P1–2 = 0.098, P2–3 = 0.206, P1–3 = 0.308, P3–4 = 0.028, P1–4 = 0.02, P2–4 = 0.976). Group 1 (n = 119), CS-negative + HBV-negative; group 2 (n = 38), CS-positive + HBV-negative; group 3 (n = 623), CS-negative + HBV-positive; group 4 (n = 166), CS-positive + HBV-positive. CS, Clonorchis sinensis; HBV, Hepatitis B virus; OS, overall survival; RFS, recurrence-free survival.

OS and RFS data for the four subgroups of patients were summarized in Fig 3 and compared with each other. There were 82 relapses and 40 deaths in the double-negative group, 23 relapses and 14 deaths in the simple CS group, 423 relapses and 210 deaths in the simple HBV group, and 114 relapses and 67 deaths in the double-positive group. The 5-year OS and RFS of the patients in the four subgroups (double-negative group, simple CS group, simple HBV group, and double-positive group) were 64.0%, 50.2%, 63.8%, 47.8%, and 22.4%, 18.5%, 19.9%, and 12.2% respectively. The median OS time and RFS time of the above four groups were 42 months and 17 months, 20 months and 7 months, 44 months and 12 months, and 27 months and 9 months, respectively. Out of the four subgroups, the prognosis of the patients in the double-positive group was considerably worse than those in the double-negative group (5-year OS: 47.8% vs. 64.0%, P = 0.012; 5-year RFS: 12.2% vs. 22.4%, P = 0.020; Fig 3). Further, the data revealed that the prognosis of the patients in the double-positive group was considerably worse than that of patients in the HBV group (5-year OS: 47.8% vs. 63.8%%, P < 0.001; 5-year RFS: 12.2% vs. 19.9%, P = 0.028; Fig 3). However, the results revealed no statistically significant differences in the 5-year OS (47.8% vs. 50.2%, P = 0.924; Fig 3) and RFS (12.2% vs. 18.5%, P = 0.976; Fig 3) between the double-positive group and the simple CS group. Thus, the data indicated that the patients in the double-positive group had the worst prognosis.

### Risk factors for survival and recurrence

The four subgroups were added along with clinicopathology information as factors to multiple Cox regression models for OS and RFS. Univariate analysis was used to analyze 20 different factors that could influence the OS or RFS in patients with HCC, including age (< 60 or ≥ 60 years), gender (male or female), the number of tumors (single or multiple), tumor size (≤ 5 cm or > 5 cm), BCLC stage (A, B or C), Edmonson grade (I-II or III-IV), MVI (positive or negative), tumor capsule (yes or no), tumor satellite focus (yes or no), macarovascular invasion (yes or no), liver cirrhosis (yes or no), serum albumin (< 35g/L or ≥ 35g/L), ALT (≤ 40U/L or > 40U/L), AST (≤ 40U /L or > 40U/L), TBil (≤ 17.1μmol/ml or ˃ 17.1μmol/ml), AFP (<400ng/ml or ≥400ng/ml), LYMPH (<1.835 x109/L or ≥1.835 x109/L), NEUT (<3.82 x109/L or ≥3.82 x109/L), EO (<0.2 x109/L or ≥0.2 x109/L), and the four subgroups (double-negative group, simple CS group, simple HBV group, and double-positive group).

In this study, twelve variables were identified as significant predictors of OS and RFS, including multiple tumor numbers, tumor size > 5 cm, Edmonson grade III-IV, MVI positivity, BCLC-C stage, tumor satellite focus positivity, macrovascular invasion, serum albumin, AST > 40 U/L, NEUT ≥ 3.82 x109/L and AFP > 400 ng/ml. The double-positive group was a significant risk factor for both OS and RFS.

Multivariate Cox analysis revealed that tumor size > 5 cm, Edmonson grade III-IV, BCLC-C stage, and tumor satellite focus positivity were independent risk factors for OS and RFS in these patients. Further, the male gender and the number of multiple tumors were identified as independent risk factors for RFS. Macrovascular invasion and EO ≥ 0.2 x10^9^/L were independent risk factors for OS (Table 3).

**Table 3:** Univariate and multivariate analysis of prognostic factors for recurrence-free survival (RFS) and overall survival (OS) among all HCC patients (n = 946).

Variable	RFS				OS			
	Univariate analysis	P	Multivariate analysis	P	Univariate analysis	P	Multivariate analysis	P
Gender(Female)	0.695(0.543–0.889)	**0.004**	0.724(0.562–0.933)	**0.013**	0.714(0.505–1.009)	0.056		
Age(≥60 years)	0.927(0.773–1.111)	0.412			0.857(0.661–1.110)	0.242		
Tumor size(>5cm)	1.552(1.323–1.820)	**<0.001**	1.299(1.095–1.541)	**0.003**	2.017(1.598–2.546)	**<0.001**	1.569(1.226–2.007)	**<0.001**
No. of tumors(multiple)	1.598(1.357–1.882)	**<0.001**	1.348(1.127–1.611)	**0.001**	1.377(1.096–1.730)	**0.006**	1.186(0.925–1.512)	0.179
Capsule of tumor(Yes)	0.854(0.704–1.036)	0.109			0.928(0.711–1.212)	0.584		
MVI(positive)	1.580(1.349–1.849)	**<0.001**	1.159(0.976–1.377)	0.093	1.757(1.414–2.182)	**<0.001**	1.200(0.947–1.519)	0.132
BCLC stage(C)	1.998(1.691–2.363)	**<0.001**	1.606(1.249–2.065)	**<0.001**	2.546(2.041–3.177)	**<0.001**	1.410(1.002–1.982)	**0.048**
Edmonson grade(III-IV)	1.570(1.342–1.837)	**<0.001**	1.426(1.211–1.678)	**<0.001**	1.786(1.431–2.229)	**<0.001**	1.584(1.262–1.989)	**<0.001**
satellite focus(Yes)	2.235(1.811–2.759)	**<0.001**	1.695(1.351–2.127)	**<0.001**	2.256 (1.718–2.961)	**<0.001**	1.626(1.207–2.191)	**0.001**
Macarovascular invasion(Yes)	1.764(1.450–2.147)	**<0.001**	0.960(0.727–1.267)	0.773	2.702(2.117–3.449)	**<0.001**	1.500(1.048–2.147)	**0.027**
Liver cirrhosis(Yes)	1.186(1.014–1.387)	**0.033**	1.071(0.907–1.264)	0.418	1.000(0.806–1.242)	0.997		
Serum albumin(≥35g/L)	0.716(0.565–0.908)	**0.006**	0.832(0.652–1.062)	0.139	0.700(0.505–0.969)	**0.032**	0.895(0.642–1.247)	0.511
ALT(>40U/L)	1.208(1.031–1.416)	**0.019**	1.064(0.883–1.282)	0.512	1.109(0.889–1.383)	0.359		
AST(>40U/L)	1.242(1.063–1.451)	**0.006**	1.069(0.888–1.287)	0.481	1.318(1.062–1.636)	**0.012**	1.119(0.896–1.398)	0.321
TBil(˃17.1μmol/ml)	0.930(0.759–1.139)	0.481			0.802(0.597–1.075)	0.140		
AFP(≥400ng/ml)	1.366(1.169–1.596)	**<0.001**	1.169(0.993–1.376)	0.061	1.491(1.202–1.851)	**<0.001**	1.155(0.922–1.448)	0.210
NEUT(≥3.82 x109L)	1.187(1.016–1.386)	**0.030**	1.057(0.896–1.247)	0.512	1.459(1.174–1.813)	**0.001**	1.196(0.953–1.501)	0.122
LYMPH(≥1.835 x109L)	0.944(0.808–1.102)	0.465			0.878(0.708–1.090)	0.238		
EO(≥0.2 x109L)	1.125(0.964–1.314)	0.136			1.406(1.131–1.748)	**0.002**	1.257(1.001–1.579)	**0.049**
CS positive and HBV negative	1.446(0.910–2.299)	0.119			1.747(0.949–3.215)	0.073		
CS negative and HBV positive	1.120(0.884–1.420)	0.347			1.031(0.735–1.446)	0.862		
Double positive	1.405(1.057–1.867)	**0.019**	1.170(0.870–1.575)	0.299	1.684(1.137–2.494)	**0.009**	1.303(0.864–1.965)	0.207

BCLC: Barcelona Clinic Liver Cancer Staging System. MVI: Microvascular invasion. HBsAg: hepatitis B surface antigen. TBil: total bilirubin. AST: aspartate aminotransferase. ALT: alanine aminotransferase. NEUT: absolute neutrophil count. EO: the absolute number of eosinophils. LYMPH: absolute lymphocyte count. CS: Clonorchis sinensis. HBV: Hepatitis B Virus

Factors associated with OS and RFS in the double-positive and simple HBV groups: In the double-positive group of patients, tumor size > 5 cm, Edmonson grade III-IV, liver cirrhosis, and tumor satellite focus, and AST > 40 U/L were independently associated with poorer RFS. In addition, only tumor size was independently associated with poorer OS in the multifactorial analysis (S1 Table).

In the simple HBV group, multiple tumors, BCLC-C stage, Edmonson grade III-IV and tumor satellite focus were independently associated with worse RFS, and tumor size >5 cm, multiple tumors, BCLC-C stage, Edmonson grade III-IV and tumor satellite focus were independently associated with poorer OS (S2 Table).

Cirrhosis was found to be independently related to poor RFS only in the double-positive group and not in the simple HBV group. By classifying cirrhosis according to the two groups, Kaplan-Meier provided additional evidence for this conclusion. Cirrhosis was not linked to the poor OS in the simple HBV and double-positive groups (S1 Fig).

## Discussion

This study aimed to evaluate the effects of CS and HBV infection separately and CS combined with HBV infection on the prognosis of HCC after radical hepatectomy. The findings demonstrated that CS as a single variable as well as combined with HBV infection, could impact the RFS and OS rates following HCC surgery. The data revealed that HCC patients with CS and HBV infection (i.e., double-positive) had a worse prognosis than HCC patients without infection (i.e., double-negative).

HBV is a major cause of HCC and can promote the development of hepatocellular carcinoma through direct and indirect mechanisms. Integrating HBV DNA into the host genome in the early stages of tumor clonal expansion causes genomic instability and insertional mutations in several cancer-related genes. Prolonged expression of the viral regulatory protein HBx leads to cellular transcription and proliferation control dysregulation, sensitizing hepatocytes to oncogenic factors. HBV-associated HCC has a high rate of chromosomal alterations, and mutations increase the rate of p53 inactivation and the overexpression rate of hepatic progenitor cell genes [24]. HBV also contributes to postoperative liver failure and tumor recurrence in patients with HCC. Previous studies have reported various indicators associated with HBV infection and affecting prognosis, such as serum HBeAg positivity [10], high HBV DNA levels, HBsAg positivity [25], and high HBsAg levels [26–28]. The serological marker HBsAg, which HBV produces, is crucial for diagnosing and monitoring patients with CHB. Preoperative serum HBsAg positivity in patients with HCC has been reported to be strongly associated with postoperative relapse [25,29]. It has also been demonstrated that preoperative HBsAg levels accurately predict long-term recurrence and survival of patients having surgical resection for HBV-related HCC [30]. Therefore, in this study, HBsAg qualitative approach was used to confirm HBV infection and group. However, all patients in the study were dichotomized by HBsAg characterization, and the differences in postoperative OS and RFS between the HBsAg-negative and positive groups of HCC patients were not statistically significant (P = 0.406/ 0.943). The reason for this result could be the biased selection of the cohort, the large proportion of HBsAg-positive patients, or the presence of patients with occult HBV infection [31]. Additionally, the individuals might not show any HBV markers despite having the infection if they have occult HBV infection [32]. Seronegativity in these patients may be due to natural mutations in HBV that alter the amount of serum HBsAg or the immunoreactivity of various HBV proteins [33].

*Clonorchis sinensis* has been confirmed as the causative agent of clonorchiasis, which poses a socioeconomic burden to the infected areas. After CS infection, the parasite migrates into the bile duct with human bile, causing a series of lesions that promote an environment of continuous oxidative stress and chronic inflammation in the bile ducts and the surrounding liver tissue. This environmental change triggers epithelial hyperplasia, periductal fibrosis, and the development of cholangiocarcinoma [34]. Statistically, 16.44% of HCC patients in China’s endemic area were also infected with CS, while 2.40% of patients without cancer were also infected with CS [35]. It was found that CS infection is a significant risk factor for the occurrence of HCC, and the risk of HCC increases with the prolongation of the course of clonorchiasis [17,35]. Therefore, much attention must be paid to the relationship between HCC and CS. The impact of CS infection on the postoperative prognosis of patients with HCC also needs to be clarified.

Through different mechanisms, CS and HBV may affect the biological characteristics of hepatocellular carcinoma and patient prognosis. The major pathogenic manifestations of CS infection are the mechanical obstruction of the bile duct caused by CS, physical damage to the bile duct epithelium by ingestion and migration, and chemical stimulation of their excretory and secretory products (ESPs) [36]. ESPs of *Clonorchis sinensis* (CsESPs) include a complex mixture of secretory proteins and some extracellular vesicles (EVs) secreted through the parasite. They could also be products from the oral or intestinal tract of the parasite. CsESPs can cause histopathological changes such as inflammation, fibrosis, and adenomatous proliferation of the bile duct epithelium. Csseverin and CsGIIIsPLA2 are both components of CsESPs, and recombinant Csseverin has been reported to inhibit apoptosis in human hepatocellular carcinoma cells significantly.

Additionally, Csseverin may exacerbate the process of CS co-infection in HCC patients by promoting apoptosis inhibition [37]. Through the protein kinase B (AKT) pathway, overexpression of CsGIIIsPLA2 can promote HCC cell migration and induce epithelial-mesenchymal transformation of HCC cells [38]. Thus, CsESP can promote the growth and dissemination of HCC cells, resulting in an unfavorable prognosis for patients. HBV infection may reorganize host genes through DNA integration, leading to host cell genomic instability and the production of oncogenic fusion proteins. HBV can induce immune responses leading to recurrent liver inflammation, fibrosis, and immune microenvironment defects. Both can cause decreased liver function or recurrence of hepatocellular carcinoma leading to poor patient prognosis.

Studies have shown that the co-infection of HBV with CS has a promoting effect. The co-infection has also been reported to inhibit the immune response by stimulating IL-10 production, inhibiting IFN-γ secretion, weakening liver function, and promoting HBV proliferation, thereby leading to fibrosis and cirrhosis [39]. Our results further demonstrate this, which reveals that cirrhosis is only independently associated with poor recurrence-free survival in the double-positive group. This may accelerate the progression of cirrhosis, which in turn causes tumor recurrence. Even though both HBV and CS infection can impair liver function, the effect of the former is more pronounced. After the co-infection, CS may assist in the replication of the HBV, causing further damage to the postoperative liver function of patients with HCC, resulting in a poor prognosis.

In this study, the double-positive group and the preoperative AFP (> 400ng/ml) were crucial predictors for RFS and OS following hepatectomy in patients with HCC, indicating that the double-positive group does affect the prognosis of patients. Macrovascular invasion is also an independent predictor of OS following hepatectomy in patients with HCC. Additionally, tumor satellite focus is an independent risk factor for OS and RFS in patients with HCC following hepatectomy. Reports indicate that tumor satellite focus are distant intrahepatic recurrence predictors and possible markers of advanced systemic disease [40]. Some studies have also reported that portal vein tumor thrombus and AFP levels are influential factors for OS and disease-free survival (DFS) [41]. These have also been verified in the present study. Notably, it has also been reported that AFP levels are an essential indicator of tumor size, which has a negligible impact on overall survival [42].

The findings of this study reveal that a combination of CS and HBV infection causes a poor prognosis for patients with HCC treated with hepatectomy. However, the study has certain limitations. First, this study used preoperative serum HBsAg characterization to confirm HBV infection and group patients. This confirmation mode was used because the year of inclusion was too large, and most patients were not tested for HBsAg and HBV DNA quantification. Thus, this study did not group the patients by HBsAg or HBV DNA quantification, and the HBV infection was determined solely by preoperative serum HBsAg characterization. Second, the cases included in this study may have biased the results due to the large year span and the advancement of medical level and strategies. Additionally, although the absence of a treatment plan for postoperative recurrence may impact the evaluation of OS, it has no bearing on the evaluation of RFS. However, we still consider this to be one of the limitations of the present study.

Further, multi-center randomized controlled studies with large sample sizes need to be performed to validate these results. Clinical diagnosis of CS includes patients with an epidemiological history of CS and corresponding imaging manifestations. Another limitation of this study is a proper clinical diagnosis of CS since diagnosing corresponding imaging manifestations is relatively tricky.

In conclusion, the findings of this study should be interpreted cautiously based on the discussed limitations. Despite these limitations, the data demonstrate that CS infection and CS co-infection with HBV can effectively assess the prognosis of patients with postoperative HCC. This information may provide more effective treatment and prolonged survival for HCC patients with co-infection with CS and HBV after hepatectomy.

## Supporting information

S1 Table Univariate and multivariate analysis of prognostic factors for RFS and OS in the double-positive group patients. (DOCX)

S2 Table Univariate and multivariate analysis of prognostic factors for RFS and OS in the simple HBV group. (DOCX)

S1 Fig Impact of cirrhosis on the prognosis following hepatectomy in the simple HBV and double-positive groups. (DOCX)

S1 Data Data file. (XLSX)
