# Supplementary material for: Effects of Clonorchis sinensis combined with Hepatitis B virus infection on the prognosis of patients with Hepatocellular Carcinoma following Hepatectomy
Source: PLoS Negl Trop Dis. 2023 Jan 13;17(1):e0011012. doi: 10.1371/journal.pntd.0011012 (PMC9879467; doi:10.1371/journal.pntd.0011012)
Supplement: S1 Table — (DOCX) [file pntd.0011012.s001.docx]

**S1 Table.** **Univariate and multivariate analysis of prognostic factors for** **recurrence-free survival (RFS) and overall survival (OS)** **in the double-positive group patients** **(n=166)**

| Variable | RFS | | | | OS | | | |
| --- | --- | --- | --- | --- | --- | --- | --- | --- |
|  | [Univariate analysis](file:///D:/Dict/8.7.0.0/resultui/html/index.html#/javascript:;) | P | [Multivariate analysis](file:///D:/Dict/8.7.0.0/resultui/html/index.html#/javascript:;) | P | [Univariate analysis](file:///D:/Dict/8.7.0.0/resultui/html/index.html#/javascript:;) | P | [Multivariate analysis](file:///D:/Dict/8.7.0.0/resultui/html/index.html#/javascript:;) | P |
| Gender(Female) | 0.975(0.309-3.078) | 0.966 |  |  | 0.511 (0.071-3.688) | 0.505 |  |  |
| Age(≥60 years) | 0.757(0.432-1.328) | 0.332 |  |  | 0.654(0.299-1.433) | 0.289 |  |  |
| Tumor size(>5cm) | 2.376(1.583-3.568) | **<0.001** | 1.935(1.239-3.022) | **0.004** | 2.771(1.590-4.829) | **<0.001** | 2.387(1.323-4.307) | **0.004** |
| No. of tumors(multiple) | 1.520(1.038-2.226) | **0.031** | 0.992(0.644 -1.529) | 0.972 | 1.195(0.721-1.980) | 0.490 |  |  |
| Capsule of tumor(Yes) | 1.308(0.770-2.222) | 0.321 |  |  | 1.353(0.690-2.655) | 0.379 |  |  |
| MVI(positive) | 1.806(1.241-2.629) | **0.002** | 1.059(0.663-1.693) | 0.810 | 1.914(1.170-3.131) | **0.010** | 1.186(0.677-2.081) | 0.551 |
| BCLC stage(C) | 1.828(1.256-2.662) | **0.002** | 1.196(0.675-2.117) | 0.540 | 2.127(1.305-3.466) | **0.002** | 1.122(0.523-2.405) | 0.767 |
| Edmonson grade(Ⅲ-Ⅳ) | 1.727(1.181-2.526) | **0.005** | 1.559(1.015-2.395) | **0.043** | 1.376(0.845-2.240) | 0.200 |  |  |
| satellite focus(Yes) | 3.187(1.956-5.192) | **<0.001** | 1.895(1.074-3.342) | **0.027** | 2.577 (1.421-4.672) | **0.002** | 1.576(0.827-3.003) | 0.166 |
| Macarovascular invasion(Yes) | 1.593(1.046-2.425) | **0.030** | 1.177(0.640-2.159) | 0.598 | 2.407(1.433-4.043) | **0.001** | 1.909(0.875-4.165) | 0.104 |
| Liver cirrhosis(Yes) | 1.555(1.036-2.334) | **0.033** | 1.580(1.008-2.474) | **0.046** | 1.563(0.914-2.674) | 0.103 |  |  |
| Serum albumin(≥35g/L) | 0.814(0.484-1.368) | 0.437 |  |  | 0.919(0.454-1.860) | 0.814 |  |  |
| ALT(>40U/L) | 1.484(1.026-2.146) | **0.036** | 0.963(0.588-1.576) | 0.881 | 1.431(0.886-2.312) | 0.143 |  |  |
| AST(>40U/L) | 1.837(1.265-2.668) | **0.001** | 1.883(1.118-3.171) | **0.017** | 1.464(0.904-2.371) | 0.121 |  |  |
| TBil(˃17.1μmol/ml) | 1.051(0.681-1.622) | 0.823 |  |  | 1.023(0.574-1.821) | 0.939 |  |  |
| AFP(≥400ng/ml) | 1.371(0.947-1.986) | 0.094 |  |  | 1.083(0.670-1.750) | 0.744 |  |  |
| NEUT(≥3.82 x109L) | 1.121(0.749-1.678) | 0.578 |  |  | 1.601(0.901-2.845) | 0.109 |  |  |
| LYMPH(≥1.835 x109L) | 0.732(0.505-1.061) | 0.099 |  |  | 0.809(0.496-1.318) | 0.395 |  |  |
| EO(≥0.2 x109L) | 0.954(0.606-1.501) | 0.838 |  |  | 0.809(0.465-1.406) | 0.452 |  |  |

BCLC: Barcelona Clinic Liver Cancer Staging System. MVI: Microvascular invasion. HBsAg: hepatitis B surface antigen. TBil: total bilirubin. AST: aspartate aminotransferase. ALT: alanine aminotransferase. NEUT: absolute neutrophil count. EO: the absolute number of eosinophils. LYMPH: absolute lymphocyte count. CS: Clonorchis sinensis. HBV: Hepatitis B Virus.
