# Supplementary material for: Effects of Clonorchis sinensis combined with Hepatitis B virus infection on the prognosis of patients with Hepatocellular Carcinoma following Hepatectomy
Source: PLoS Negl Trop Dis. 2023 Jan 13;17(1):e0011012. doi: 10.1371/journal.pntd.0011012 (PMC9879467; doi:10.1371/journal.pntd.0011012)
Supplement: S2 Table — (DOCX) [file pntd.0011012.s002.docx]

**S2** **Table**. **Univariate and multivariate analysis of prognostic factors for** **recurrence-free survival (RFS) and overall survival (OS)** **in the** **﻿simple HBV group (n=623)**

| Variable | RFS | | | | OS | | | |
| --- | --- | --- | --- | --- | --- | --- | --- | --- |
|  | [Univariate analysis](file:///D:/Dict/8.7.0.0/resultui/html/index.html#/javascript:;) | P | [Multivariate analysis](file:///D:/Dict/8.7.0.0/resultui/html/index.html#/javascript:;) | P | [Univariate analysis](file:///D:/Dict/8.7.0.0/resultui/html/index.html#/javascript:;) | P | [Multivariate analysis](file:///D:/Dict/8.7.0.0/resultui/html/index.html#/javascript:;) | P |
| Gender(Female) | 0.778(0.587-1.030) | 0.080 |  |  | 0.831(0.561-1.230) | 0.355 |  |  |
| Age(≥60 years) | 1.017(0.809-1.278) | 0.886 |  |  | 0.896(0.642-1.250) | 0.517 |  |  |
| Tumor size(>5cm) | 1.428(1.175-1.736) | **<0.001** | 1.184(0.966-1.452) | 0.104 | 1.935(1.449-2.585) | **<0.001** | 1.537(1.127-2.096) | **0.007** |
| No. of tumors(multiple) | 1.585(1.294-1.940) | **<0.001** | 1.450(1.175 -1.789) | **0.001** | 1.492(1.123-1.983) | **0.006** | 1.361(1.008-1.836) | **0.044** |
| Capsule of tumor(Yes) | 0.790(0.623-1.001) | 0.051 |  |  | 0.908(0.649-1.270) | 0.573 |  |  |
| MVI(positive) | 1.505(1.239-1.829) | **<0.001** | 1.210(0.986-1.485) | 0.067 | 1.784(1.359-2.343) | **<0.001** | 1.291(0.967-1.722) | 0.083 |
| BCLC stage(C) | 1.980(1.595-2.457) | **<0.001** | 1.690(1.206-2.369) | **0.002** | 2.711(2.045-3.593) | **<0.001** | 1.640(1.049-2.566) | **0.030** |
| Edmonson grade(Ⅲ-Ⅳ) | 1.531(1.262-1.858) | **<0.001** | 1.431(1.177-1.740) | **<0.001** | 1.932 (1.458-2.559) | **<0.001** | 1.740(1.307-2.318) | **<0.001** |
| satellite focus(Yes) | 2.059(1.583-2.679) | **<0.001** | 1.602(1.214-2.114) | **0.001** | 2.520 (1.805-3.519) | **<0.001** | 1.680(1.176-2.399) | **0.004** |
| Macarovascular invasion(Yes) | 1.894(1.479-2.426) | **<0.001** | 0.998(0.685-1.452) | 0.990 | 2.794(2.053-3.803) | **<0.001** | 1.338(0.829-2.160) | 0.234 |
| Liver cirrhosis(Yes) | 1.149(0.948-1.394) | 0.157 |  |  | 0.913(0.696-1.198) | 0.510 |  |  |
| Serum albumin(≥35g/L) | 0.764(0.570-1.022) | 0.070 |  |  | 0.655(0.441-0.972) | **0.036** | 0.741(0.496-1.107) | 0.144 |
| ALT(>40U/L) | 1.236(1.017-1.503) | **0.033** | 1.112(0.912-1.355) | 0.295 | 1.157(0.878-1.524) | 0.300 |  |  |
| AST(>40U/L) | 1.186(0.979-1.435) | 0.081 |  |  | 1.329(1.013-1.745) | **0.040** | 1.039(0.781-1.383) | 0.792 |
| TBil(˃17.1μmol/ml) | 0.891(0.685-1.160) | 0.391 |  |  | 0.721(0.487-1.067) | 0.102 |  |  |
| AFP(≥400ng/ml) | 1.383(1.142-1.674) | **0.001** | 1.200(0.987-1.460 | 0.068 | 1.542(1.176-2.022) | **0.002** | 1.236(0.934-1.635) | 0.138 |
| NEUT(≥3.82 x109L) | 1.197(0.988-1.450) | 0.066 |  |  | 1.380(1.053-1.809) | **0.020** | 1.117(0.841-1.485) | 0.444 |
| LYMPH(≥1.835 x109L) | 0.948 (0.782-1.149) | 0.584 |  |  | 0.825(0.627-1.084) | 0.168 |  |  |
| EO(≥0.2 x109L) | 1.054(0.870-1.278) | 0.588 |  |  | 1.362(1.039-1.785) | **0.025** | 1.184(0.897-1.562) | 0.233 |

BCLC: Barcelona Clinic Liver Cancer Staging System. MVI: Microvascular invasion. HBsAg: hepatitis B surface antigen. TBil: total bilirubin. AST: aspartate aminotransferase. ALT: alanine aminotransferase. NEUT: absolute neutrophil count. EO: the absolute number of eosinophils. LYMPH: absolute lymphocyte count. CS: Clonorchis sinensis. HBV: Hepatitis B Virus.
