# Supplementary material for: Effects of Clonorchis sinensis combined with Hepatitis B virus infection on the prognosis of patients with Hepatocellular Carcinoma following Hepatectomy
Source: PLoS Negl Trop Dis. 2023 Jan 13;17(1):e0011012. doi: 10.1371/journal.pntd.0011012 (PMC9879467; doi:10.1371/journal.pntd.0011012)
Supplement: S1 Fig — (DOCX) [file pntd.0011012.s003.docx]

**S1 Fig. Impact of cirrhosis on the prognosis following hepatectomy in the simple HBV and** **double-positive groups.**


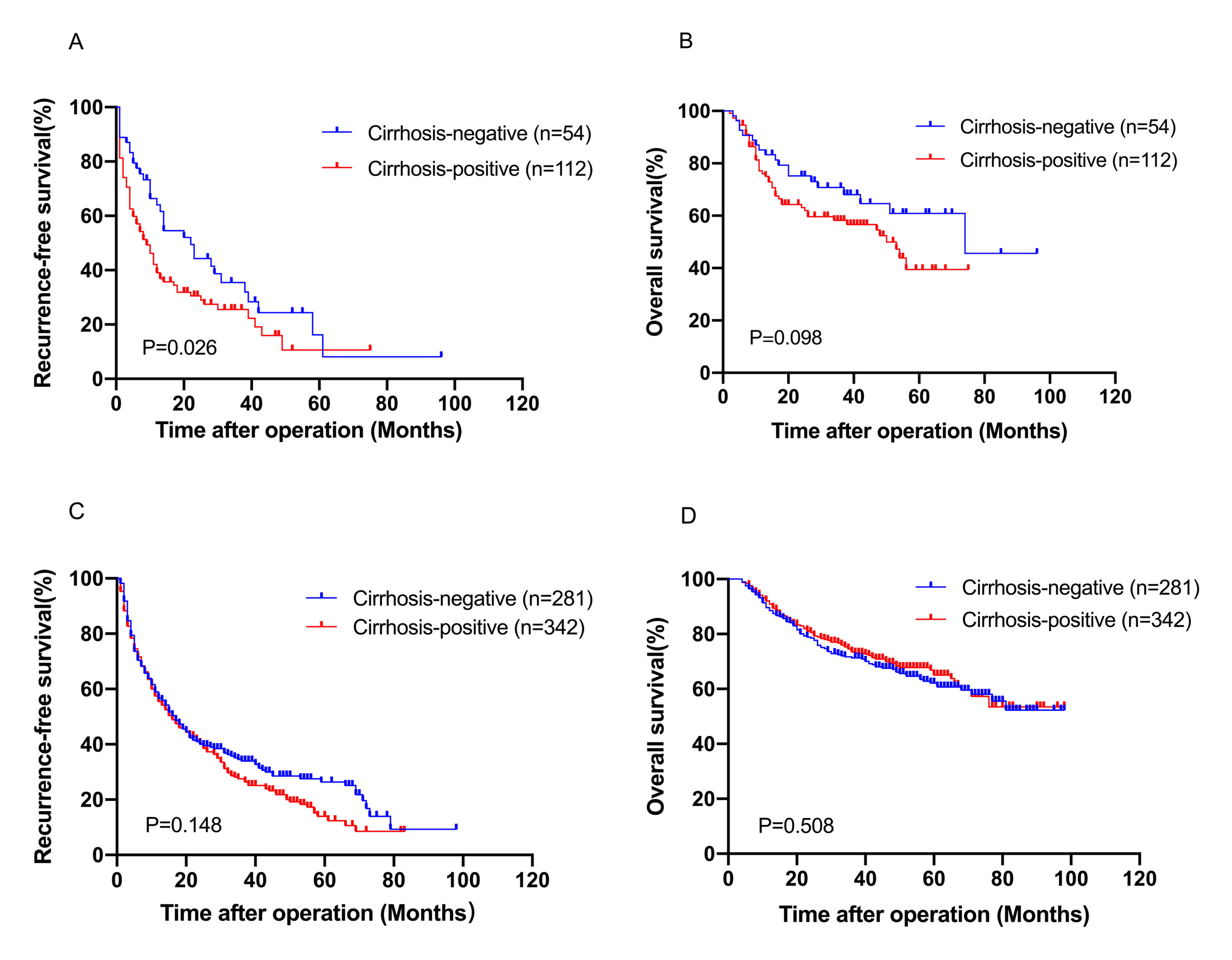


**(A),** **Recurrence-free survival for** **the double-positive group; (B), Overall survival for the double-positive group; (C),** **Recurrence-free survival for** **the simple HBV group; (D), Overall survival for the simple HBV group**
